# Carbon-Based Nanomaterials 4.0

**DOI:** 10.3390/ijms25053032

**Published:** 2024-03-06

**Authors:** Ana M. Díez-Pascual

**Affiliations:** Departamento de Química Analítica, Facultad de Ciencias, Química Física e Ingeniería Química, Universidad de Alcalá, Ctra, Madrid-Barcelona Km. 33.6, Alcalá de Henares, 28805 Madrid, Spain; am.diez@uah.es

Research on carbon-based nanomaterials, such as carbon nanotubes and graphene and its derivatives, has experienced exponential development in recent years. The unlimited possibilities to adjust and tailor carbon nanomaterials are related to their nanometer size and huge specific surface area, making them suitable for a wide range of applications. The intention of this Special Issue is to provide an opportunity for the publication of articles regarding carbon-based nanomaterials and their uses in different fields, such as electronics, energy storage, biomedicine, and sensing.

Electrospinning is a technique widely used for processing carbon nanotubes and their corresponding polymeric nanocomposites [1,2]. The main benefit of this approach is the possibility to control the fiber diameter and pore size via modifying the setup parameters [3]. However, this technique cannot be applied to some composites due to their solution viscosity or low conductivity [4,5,6]. The incorporation of CNTs is an easy way to control the rheological properties of polymer solutions, although the effects are strongly dependent on the nanotube size, concentration and state of dispersion [7,8]. When CNTs are mixed with an insulating matrix, they form a network that increases matrix conductivity [9]. The electrical properties of CNTs also lead to their alignment within the scaffold during electrospinning, unlike when they spread on polymer films, which builds a different kind of network [10,11].

Graphene oxide (GO) is an oxidized form of graphene that can bring numerous benefits, including functionalization capability, amphiphilicity, and biocompatibility [12], and can be suitable as sensitive material for small-dimension dosimeters. These GO-based dosimeters can deliver stable readings and the 3D spatial distribution of a dose, information which is decisive in areas such as radiotherapy and radioactive contamination. Several studies have reported the feasibility of GO as a dosimeter using different types of ions [13,14], and it was found that without electronic power, GO reduction was proportional to the absorbed dose, while after power was restored, the linearity vanished.

In addition, nanosized rGO spots can be produced via the swift heavy ion (SHI) bombardment of GO films. These spots can be regarded as graphene quantum dots surrounded by an insulating matrix at low doses of irradiation [15]. They comprise oxygenated groups at the edges that can act as reaction sites and modify the photoluminescence emitted from the dots by varying their electron density [16]. Their synthesis via radiation technology is an effective, rapid, and scalable method for the preparation of graphene quantum dots that enables one to adjust morphology and size [17]. Cutroneo et al. [18] recently reported the synthesis of nanosized GO spots at low doses of ion irradiation in a regime of electronic stopping power. These nanostructures show great potential to be used in applications requiring huge area coverage like light-controlled conductive switching [19].

The use of renewable clean energies is a key way to globally attain net-zero emissions in 2050 [20,21]. With the continuous progress of renewable energy sources, very stable energy storage devices are needed for leveling the intermittent electricity output [22]. Most operational projects use lithium- or sodium-ion batteries as substitutes for ESDs [23] due to their rapid response time and tailorable output. Sodium-ion batteries seem to be preferable since the element is cheaper and more abundant [24]. However, the execution of this technology has been delayed due to several issues, including the kinetic mismatch between the anode and cathode. Carbon nanomaterials have emerged as potential anodes for this type of battery owing to their improved electrical, thermal, and mechanical properties and cost-effectiveness [25,26]. Among them, microporous carbon has been demonstrated to be a good candidate [27]. In this regard, nitrogen-doped and zinc-confined microporous carbon particles were recently prepared via thermally pyrolyzed polyhedral ZIF-8 nanoparticles which were used as anodes in sodium-ion batteries/capacitors [28].

Depression is a common disorder that affects people all over the world [29]. Consequently, many efforts are devoted to the design of novel drugs that aid patients in overcoming this illness. Vortioxetine (VOR) is one of the most common drugs used for this type of treatment [30,31]. It employs a multimodal means of action via changing the activity of serotonergic receptors and inhibiting the activity of serotonin transporters [32]. Numerous analytical approaches have been described to attain a sensitive determination of VOR in different matrices, the most common being high-performance liquid chromatography (HPLC) [33,34,35,36]. Nonetheless, despite their good sensitivity and selectivity, they require expensive equipment and harmful organic solvents. Thus, voltametric methods may be an alternate method for VOR determination. In this regard, Smajdor et al. [37] designed a new approach based on square-wave voltammetry to detect this analyte, using glassy carbon electrodes modified with electrospun carbon nanofibers and NiCo nanoparticles, and applied it in several samples including urine and plasma.

Pathogens are key causes of infections all over the world that affect human health [38]. Usually, they are treated via radiation or chemical disinfectants [39] which are inexpensive but require high doses and generate many byproducts [40,41]. The COVID-19 pandemic has shown the necessity for antiviral nanomaterials which can be implanted in personal protective equipment [42]. Metal nanoparticles and metal complexes are also useful as antimicrobial agents due to their exceptional properties, including their nanoscale size and large specific surface area for improved interaction [43,44]. Carbon nanomaterials have also been demonstrated to be good antiviral agents. However, few works on the antiviral properties of hybrid nanomaterials have been reported. Recently, the antibacterial properties of CNTs using *E. coli* and *G. stearothermophilus strains* and the antiviral properties of different functionalized CNTs, were reported [45]. The strong physical and chemical interactions between CNTs and metal oxides can result in synergistic effects that improve antiviral efficiency [46].
